# Toxic Releases and Risk Disparity: A Spatiotemporal Model of Industrial Ecology and Social Empowerment

**DOI:** 10.3390/ijerph120606300

**Published:** 2015-06-02

**Authors:** Hannah Aoyagi, Oladele A. Ogunseitan

**Affiliations:** 1 School of Social Ecology, University of California, Irvine, CA 92697, USA; E-Mail: haoy461@ecy.wa.gov; 2 Department of Population Health and Disease Prevention, Program in Public Health, University of California, Irvine, CA 92697, USA

**Keywords:** disparity, empowerment, environment, exposure, health, industry, model, pollution, spatial analysis, toxic chemicals

## Abstract

Information-based regulations (IBRs) are founded on the theoretical premise that public participation in accomplishing policy goals is empowered by open access to information. Since its inception in 1988, the Toxics Release Inventory (TRI) has provided the framework and regulatory impetus for the compilation and distribution of data on toxic releases associated with industrial development, following the tenets of IBR. As TRI emissions are reputed to disproportionately affect low-income communities, we investigated how demographic characteristics are related to *change* in TRI emissions and toxicity risks between 1989 and 2002, and we sought to identify factors that predict these changes. We used local indicators of spatial association (LISA) maps and spatial regression techniques to study risk disparity in the Los Angeles urban area. We also surveyed 203 individuals in eight communities in the same region to measure the levels of awareness of TRI, attitudes towards air pollution, and general environmental risk. We discovered, through spatial lag models, that changes in gross and toxic emissions are related to community ethnic composition, poverty level, home ownership, and base 1989 emissions (R-square = 0.034–0.083). We generated a structural equation model to explain the determinants of social empowerment to act on the basis of environmental information. Hierarchical confirmatory factor analysis (HCFA) supports the theoretical model that individual empowerment is predicted by *risk perception*, *worry*, and *awareness* (Chi-square = 63.315, *p* = 0.022, df = 42). This study provides strong evidence that spatiotemporal changes in regional-scale environmental risks are influenced by individual-scale empowerment mediated by IBRs.

## 1. Introduction

Successful models of sustainable industrial development demand rigorous balancing of aggressive actions to maximize economic productivity and penetrating vigilance to minimize adverse impacts of industrial activity on ecosystems and public health. Information-based regulation (IBR) is an essential tool for maintaining such balances, representing one of the hallmarks of modern environmental policy in the U.S., and a symbolic shift towards public empowerment in pollution control. Pollution issues can be framed as a conflict between industries and one or more stakeholders, where industry’s access to production data creates an information asymmetry that promotes opportunistic, profit maximizing behavior at the expense of environmental quality in communities [1]. While the U.S. government has traditionally sought to protect environmental quality and human health by acting as the representative stakeholder for the public, IBRs, are designed to reduce information costs, giving the public more power to act as self-representing stakeholders to negotiate with both industry and policy-makers.

The Toxics Release Inventory (TRI) is one of the most prominent IBRs in the U.S., and is widely considered to be one of the most successful environmental regulations in the U.S. [2]. Under TRI, certain categories of industrial facilities, with 10 or more employees, manufacturing or processing over a threshold amount of any criteria chemicals, must submit an annual report to the U.S. Environmental Protection Agency (EPA). The raw data are then made available to the public, through EPA’s TRI Explorer website or directly from their offices. This listing is the extent of regulatory obligation. What is to happen next is more nebulous, and the following steps are left up to the data users, assumed to be primarily impacted communities and participating industries. As EPA states,

“Armed with TRI data, communities have more power to hold companies accountable and make informed decisions about how toxic chemicals are to be managed. The data often spurs companies to focus on their chemical management practices since they are being measured and made public.”.

[3]

In principle, TRI should correct information asymmetry by providing all stakeholders with firms’ pollution data. The idea is that by having access to pollution information, the public will become empowered to participate in implementing the policy process to reduce risks from chemical pollutants. We can call this outcome *stakeholder empowerment*. Yet, what are the units of measuring empowerment and how much of it is needed to actually result in tangible benefits to society? Empowerment became a popular political phrase in the 1990s, prompting social scientists to more critically evaluate empirical evidence of its practical applications [4]. Stakeholder empowerment is, however, still loosely defined and unstudied in the context of TRI.

TRI has recently come under scrutiny for the economic burden it places on firms and for the administrative costs to USEPA [5], and a proposal is currently being considered to reduce the program’s cost to both the agency and reporting entities by requiring reporting on a biennial rather than an annual basis [6]. What is missing from the discussion, however, is an evaluation of how TRI has fulfilled its programmatic goal of stakeholder empowerment, or if it has done so selectively, leaving a pattern of risk disparity across communities. If TRI is designed to empower communities and the data are indeed being utilized, then we might expect to see a weaker relationship between socioeconomic status and TRI exposures, as people with the least initial access to toxics information should experience the greatest gains from such a program. The more realistic scenario is that those disempowered communities still lack the resources to utilize the data, and without more proactive policies, we should expect to see a worsening of the problem as more empowered neighborhoods are able to pressure firms to move or reduce pollution. Accounting for the phenomenon of spatial clustering seems to improve the predictive power of demographic variables in determining TRI facility proximity and emissions exposures [7], and so we precede our regression analysis with a descriptive inquiry into the spatial patterning of TRI locations and emissions, from 1989 to 2002. If TRI does tend to better help communities with more resources, then we expect to see stronger spatial patterns in 2002 as a result of uneven pressures on facilities to reduce pollution in the more affluent, politically dominant neighborhoods.

We selected Los Angeles for this study because it is one of the world’s centers of diversity in terms of industrial activities and socioeconomic parameters. We found that toxic emissions from TRI facilities were slightly more clustered in 2002 than they were in 1989. Statistical regression models indicate that reductions in the emission of Hazardous Air Pollutants (HAP) between 1989 and 2002 were correlated with proportion of Hispanic population, poverty rates, and baseline 1989 emissions. Further, we identify some measurable outcomes of stakeholder empowerment, specifically individual attitudes, perceptions, and awareness. We then construct a theoretical model for stakeholder empowerment and use empirical data to support this model, using hierarchical confirmatory factor analysis (HCFA). This model links individual perception of environmental risks and the ability to identify information sources in explaining stakeholder empowerment attributable to TRI effectiveness.

## 2. Methods

### 2.1. Spatiotemporal Study of Risk Disparity

Assessments of environmental risk, its perception and disparity pose several methodological issues, in the selection of appropriate geographic scale, demographic categories, and outcome measures. These assessments are complicated because they attempt to correlate two very different classes of datasets: U.S. Census data and environmental information databases. Detailed census data are packaged in artificially-defined units of analysis (zip codes, tracts, *etc.*), whereas environmental information is much less comprehensive and not always categorized by consistent geographical units. We focus first on the nature of spatial clustering; using local indicators of spatial association (LISA) and Moran’s I [8]. Spatial clustering of environmental risk and changes in ecologically-defined exposure over time allows us to characterize the differential effects of TRI, or social and economic influences on toxic emissions levels. Second, we use ordinary least squares (OLR) and spatial regression to compare the relationship between changes in TRI exposure over time and in sociodemographics. We used a spatial coincidence method in which exposure data are aggregated by tract and joined to the corresponding census information [9,10].

#### 2.1.1. TRI Data

TRI data come with caveats. First, TRI is a self-reporting program with limited enforcement opportunities [11]. There is evidence of under-reporting, and many of the emissions reductions noted in the early 1990s were attributable to paperwork changes [12,13]. We expected some inaccuracies in the initial reporting year and accounted for possible over- or under-reporting by using 1989 for the initial emissions values. Second, TRI has a continuously evolving list of criteria chemicals and reporting thresholds, changes in which can mask the true trends in emissions over time. We chose hazardous air pollutants (HAPs) because of their documented effects on human health, and their persistence over time in the list TRI chemicals. Third, TRI has an approximate 3-year lag time between the reporting and the date that data are released to the public. We included all reportable HAPs, of which there are 190 chemicals in the TRI database [14,15]. Finally, TRI covers a subset of air pollutants from large stationary sources, excluding non-point sources of vehicle emissions and numerous domestic sources.

#### 2.1.2. Geographical Considerations

Los Angeles (LA) has one of the highest concentrations of TRI facilities in the U.S., and the region has difficulty meeting Clean Air Act goals [16]. The population in LA is exposed to high levels of criteria air pollutants, and previous studies have examined linkages between urbanization, population density, and patterns of exposure to pollutants [17,18,19]. We focused on the U.S. Census-defined Los Angeles urban area, which eliminates the less populated portions of the county while maintaining the vast majority of TRI sites. We selected census tracts as the spatial unit of analysis because they are small enough to meaningfully correlate exposure with demographics, while being sufficiently large to allow reasonable interpretation of global spatial statistics [20,21,22,23].

#### 2.1.3. Outcome Measure

We examined two types of outcomes: total emissions, and toxicity-weighted emissions of HAPs. We used toxicity-weighted total HAP emissions to better assess the health impacts of toxic emissions on surrounding communities. Each chemical has assigned chronic, non-cancer inhalation reference concentration (RfC) or a reference exposure level (REL) in mg/m^3^ [24]. In some rare cases, we used RfCs or REL for chemical groups, or minimum risk levels (MRLs), a default value of 1 mg/m^3^ (dibutyl and dimethyl phthalates were classified with di(2-ethylhexyl) phthalate). The resulting toxicity-weighted emissions values approximate the residual health risk from TRI facilities, and highlight areas of concern that might not stand out in an analysis of facility locations or raw HAP emissions data.

#### 2.1.4. Model Inputs

Pastor *et al.* [7] described three categories of environmental exposure disparities: land use (population density, urban classification); market dynamics (income, poverty, housing prices); and empowerment (ethnicity, home ownership, immigration). Of these, ethnicity is perhaps most consistently correlated with exposure, although specific ethnicity seems to interact with economic poverty [11]. Hispanics represent the largest minority population in LA, prompting us to focus on community proportion of Hispanics as a key ethnicity factor.

The relationship between poverty and the distribution of environmental hazards is not easily predictable, haven been found to be non-significant with respect to cancer risk [24], TRI toxicity, TRI releases [16], and blood lead level [25]. Other studies, however, found positive correlations with exposure to persistent bioaccumulative toxins [7], TRI facility proximity and TRI facility density [10]. Median household income is an even trickier variable, in part due to observations of a curvilinear relationship with environmental exposures, where both urban low and high income areas are more highly exposed than middle income populations [9]. Still, income is useful, as it can differentiate between middle and high income tracts that both have low poverty rates. Home ownership can be taken as an alternate representation of economic power, as well as a proxy for community stability and political power.

The simplest indicator of land use is population density and urbanization. Industrial sites tend to be clustered in parts of LA as a result of local zoning decisions and proximity to shared resources such as transportation networks [26].

#### 2.1.5. Statistical Methods

We used descriptive spatial analyses to explore and compare patterns in HAP emissions and total toxicity between the 1989 and 2002 reporting years. The *GeoDa 095i* program was used to generate local indicators of spatial association (LISA) cluster maps and Moran’s scatterplots to test for significant spatial clustering in TRI exposures [8]. We also ran ordinary least squares and spatial regressions in *GeoDa 095i* to test our theoretical models for change in HAP emissions and total toxicity between 1989 and 2002.

### 2.2. Risk Perception and Social Empowerment

Responses from 203 individuals were received from eight LA communities, between March and April of 2005. The communities were selected based on proximity to TRI facilities emitting HAPs in the 2002 reporting year, four experiencing emissions declines between 1989 and 2002, and four experiencing increases in emissions. Census block group demographic variables (percent white, income, education, and percent home-ownership) were used as predictors of the community’s collective ability to respond to TRI data, so that selected sites could also represent demographic diversity. About 2000 potential participants were randomly selected from an address database for the Census block groups that comprised the study sites. Each received a mailing with the survey materials, a cover letter explaining the study, and a card to request survey results. A second batch of mailers was sent to non-responders with an incentive, a chance to win one of five $50 gift cards. Overall, the response rate was 9.1%.

#### Survey Composition

The survey instrument consists of four sections totaling 64 questions, and solicited responses on a general framework of environmental risk perception, followed by questions specific to air pollution awareness and knowledge/use of TRI. The first section consists of an environmental risk perception scale [27], adapted from interview format to a written survey. The scale elicits respondents’ beliefs about environmental risks by having them indicate their level of agreement with a series of statements using a Likert scale. Although this measure was designed to create a composite rating, the analysis presented here focuses on respondents’ views of *trust*, *fairness*, and perception of *control* regarding environmental risks.

The second and third sections examine respondents’ perceptions of air pollution and their awareness and use of TRI, respectively. Respondents were asked to identify any local sources of air pollution they were aware of, and then to indicate how they found out about the sources. Subsequent questions solicit opinions regarding health risk from local air pollution and respondents’ perceived ability to find further information on air pollution and its health impacts. The third section explores whether or not respondents are familiar with TRI, right-to-know, or other ways to “find out how much and what type of chemicals a firm releases into the environment”. Respondents who were aware of one or more of the information sources then answered a series of questions regarding where they had heard of TRI, whether they had viewed the data, their opinions regarding the ease of accessing and interpreting the data, and whether TRI had prompted them to take any action (look for more information, tell a friend, contact a government agency, *etc.*).

The final section covers basic demographic information, from gender and ethnicity, to income, education, and whether they own their home. It was important to investigate a range of factors that might influence individual perceptions of environmental risk, and initiative to respond to TRI.

### 2.3. Survey Data

#### 2.3.1. Participants

Of the 203 respondents, 52.2% were female, with a median age of 52 years (mean = 51, range = 20 to 92). Participants ranged by ethnicity (40.4% Caucasian, 27.4% Latino, 19.7% African American, 13.6% Asian American), and by socioeconomic status, from a monthly income of $500–999 to over $4000 (mean ~$3000). Education level varied widely, with an average achievement of “some college”, and 89.7% of respondents listed English as the language they were most comfortable speaking. 62.4% reported owning their home. We believe the response rate did not unduly influence the diversity of samples, as a comparison of ethnic composition and income between the survey respondents and the base populations indicated no consistent selection bias across the groups.

#### 2.3.2. Data Transformation and Statistical Analyses

Several variables were logarithmically transformed after they were found to be non-normally distributed. Linear transformations were used to more closely align the variances within the data set. Finally, select variables were reverse-coded to obtain positive covariances among variable groupings. The *Amos 5.0.1* program maximum likelihood estimation was used to estimate the structural equation models in this analysis.

## 3. Results and Discussion

### 3.1. Spatial Data Analyses

#### 3.1.1. Local Patterns in Hazardous Air Pollutant (HAP) Releases

The number of TRI facilities reporting HAP emissions in LA declined from 542 in 1989 to 220 in 2002, with a concomitant decrease of 11.5 million kg of emissions in that period. We observe an increase in overall spatial clustering between 1989 (I = 0.081) and 2002 (I = 0.093). There is a weak temporal *change* in spatial pattern (I = 0.065), and the reduction in HAP emissions is not distributed evenly. Southeast LA, Torrance, and Wilmington—areas with high concentrations of TRI facilities—show significant clustering of decreases in emissions, which could be attributed to the notion that those areas have the most “room for improvement”. The areas of most concern are near the Port of Los Angeles/Long Beach, and in Pasadena, where emissions have increased locally. Pasadena’s cluster turns out to be based on a single facility, whose releases represent the only emissions in an area that used to have several TRI facilities. The Port area and other scattered locations, however, have experienced increases in emissions and potentially more toxic exposures, and thus we look to a more specific indicator of risk, toxicity-weighted TRI emissions.

#### 3.1.2. Toxicity-Weighted Emission Patterns

**Figure 1:**
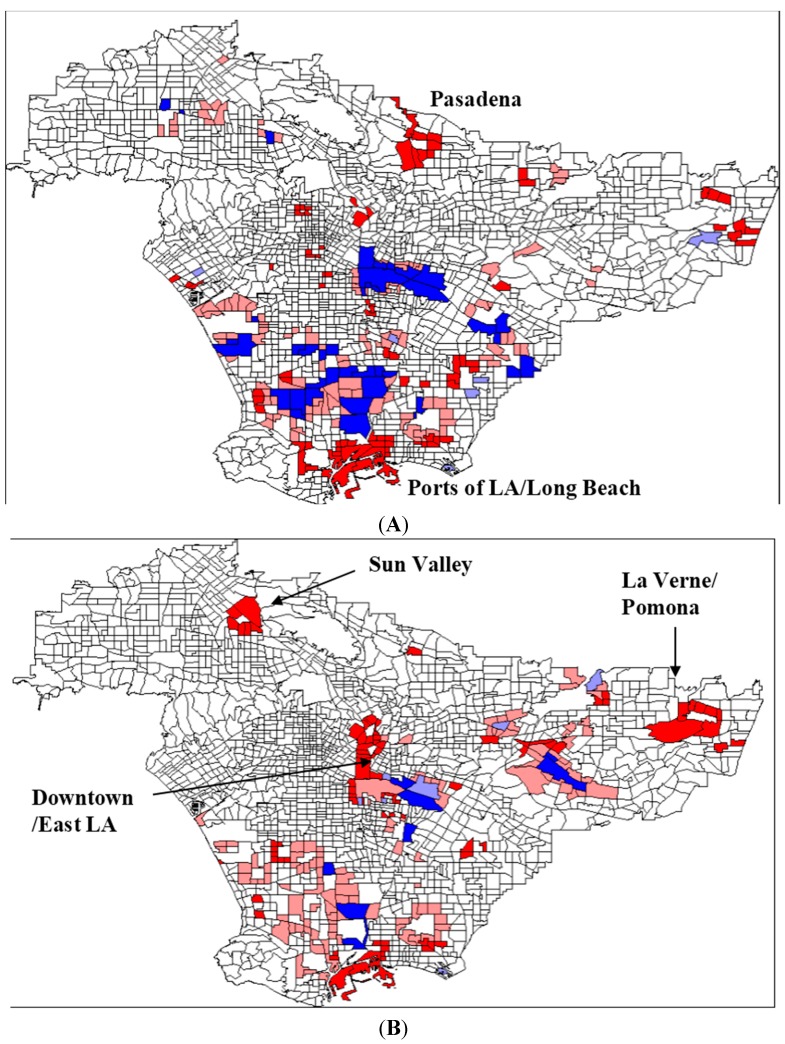
(**A**) Change in HAPs 1989–2002, I = .0653 (**B**) Change in Toxicity-Weighted HAP Releases 1989–2002, I = 0.027. Bright red areas represent census tracts with an increase in HAP emissions, surrounded by other census tracts with increased HAP emissions (“high-high”), statistically significant at the 0.05 level. Pale red areas represent census tracts with increased HAP emissions, surrounded by decreased HAP emissions (“high-low”). Pale blue areas represent census tracts with decreased HAP emissions, surrounded by increased HAP emissions (“low-high”). Bright blue areas represent census tract with decreased HAP emissions, surrounded by decreased HAP emissions (“low-low”).

Weighting each chemical emission by its relative non-cancer toxicity provides a more detailed picture of community exposure and the potential for health problems. We find that the spatial pattern is again stronger in 1989 (I = 0.035) than in 2002 (I = 0.013), although both statistics are quite low. This is likely due to the fact that the weighting process drastically widens the range of emissions values, as some HAPs can be 100,000 times more toxic than the baseline (1 mg/m^3^). Looking at the patterns in toxicity from 1989 to 2002, Southeast LA and Carson experience clusters of declines in toxicity, just as with HAP emissions, even while their overall toxicity-weighted emissions remain high (Figure 1). Again, these may represent the areas with the most room for improvement, as the area experienced an overall decline of 2.95 billion toxicity-weighted kilograms. The areas that experience clusters of increases in toxicity are the Ports of LA and Long Beach, as well as the La Verne/Pomona area, Sun Valley, and Downtown and East LA. The overall distribution of toxicity exhibits less spatial autocorrelation (I = 0.027) than the distribution of unweighted HAP emissions (I = 0.065), indicating that the potential reductions in human health risk from TRI emissions are more evenly distributed than the raw emissions data might suggest.

Overall, as the outcome measure becomes more specific, from mass HAP emissions to specific toxicity, spatial clustering becomes less pronounced, and exposures appear to be more evenly distributed. This relationship supports the notion that the more specific the measure of exposure, the less its distribution will be explained by spatial location, and the more it will potentially be influenced by social, economic, political, or other factors (When we look at the regression models in the second part of the analysis, we see that the spatial lag term in for toxicity-weighted emissions (coefficient = −0.049, *p* < 0.05) plays less of a role than the lag term for HAP emissions (coefficient = 0.246, *p* < 0.01).

### 3.2. Demographic Predictors of Change in TRI Exposures

We constructed a simple regression model for the relationship between TRI exposure (total HAP emissions and toxicity-weighted emissions) and socio-demographic factors, to test for potential differences in patterns of risk disparity from 1989 to 2002. We extended the three variable categorizations from Pastor *et al.* [7], selecting percent Hispanic and percent home-owners to represent *empowerment*, and median household income and percent living below the poverty for *economic dynamics*. The third category, *land use*, is more difficult to account for. Although population density has been used as a proxy for the clustering of facilities observed in industrial areas and should theoretically explain auto-correlated error terms [8], in our case it becomes unclear whether a spatial model is appropriate. Instead, we utilized the air emissions in 1989 and the log of toxicity-weighted emissions in 1989 to account for industrial land use, based on our observation that approximately the same tracts have TRI facilities in both years. In the case of both gross and toxicity-weighted emissions, the error terms exhibit strong autocorrelation and the regression diagnostics suggest the incorporation of a spatial lag term. In each case, we arrive at a spatial lag model that best illustrates the relationship between community characteristics and the benefits they experience regarding decreases in TRI emissions.

#### 3.2.1. Changes in Mass HAP Emissions

The real benefit of mass reductions in TRI emissions should be reflected in reduced exposure to toxic materials. However, it may sometimes be easier for an industry to reduce TRI emissions by switching to more toxic, but lower volume chemicals. To explore spatiotemporal changes in mass and/or toxicity-weighted emissions, we proposed a spatial lag model, where the lag term captures unmeasured effects related to the pattern in the location of facilities with changes in emissions. TRI facilities may decrease their emissions if neighboring industries appear to be responding to community pressures to improve their environmental image.

The ordinary least squares (OLS) model is weak (R-squared = 0.024), and the diagnostics are suggestive of a spatial lag model (Robust LM = 6.706, *p* < 0.05). The spatial lag model better predicts change in emissions (R-squared = 0.083), but does not represent a large portion of the variance (Table 1). This model accounts for 29.9% of the variance in emissions changes, where emissions increases are positively correlated with high poverty and low emissions in 1989. Interestingly, high proportion of Hispanics is correlated with emissions *decreases*, possibly as a result of their already high exposures to TRI emissions (the correlation between percent Hispanic and the log of 1989 emissions toxicity is 0.130 (*p* < 0.05).

**Table 1:** Ordinary Least Squares (OLS) and Spatial Regressions for Change in TRI HAP Emissions from 1989 to 2002.

Variables	OLS		Spatial Lag 1		Spatial Lag 2	
	Coefficient	T-Stat	Coefficient	T-Stat	Coefficient	T-Stat
**Percent Hispanic**	−0.921	−6.681 ***	−0.612	−4.531 ***	−0.527	14.413 ***
**Percent in Poverty**	1.379	4.854 ***	0.949	3.433 ***	0.607	2.495 **
**W_log of Air Emissions**			0.309	9.207 ***	0.246	7.985 ***
**N**	1942		1942		1942	
**R^2^**	0.024		0.083		0.299	

*** significant at the 0.01 level; ** significant at the 0.05 level.

#### 3.2.2. Changes in Toxicity-Weighted HAP Emissions

If decreases in emissions toxicity occur due to TRI’s effectiveness in enhancing community environmental activism, it is important to capture the characteristics exhibited by such communities. Thus, we posited that decreases in emission toxicity are related to economic income, proportion of minority population (specifically Hispanics in LA), home ownership, and to the 1989 baseline emissions toxicity. This model also requires a spatial lag term. We do not assume, however, that changes in toxicity depend as much on one’s neighbor’s actions, as decreases in toxicity are not as obvious in evaluating TRI data as reductions in mass air emissions.

Again, the initial OLS model is very weak (*R*^2^ = 0.017) pointing to a spatial lag model (Robust LM = 11.783, *p* < 0.05). Demographics account for only 4.7% of the variability in the first spatial model, but when we include baseline data for 1989 emissions toxicity, the variable overwhelms the effect of demographic factors (Table 2) (The R-squared for 1989 toxicity alone is 0.716, as compared to 0.720 for the entire model). However, the proportion of Hispanics in the community is positively correlated with emissions increases when accounting for 1989 toxicity, and negatively correlated when just looking at demographic variables. Median household income tends to be higher in areas that experience emissions *increases*, while the proportion of homeowners is lower. The direction of median household income is possibly a result of the U-shaped relationship typically seen between income and environmental exposure [8], and observed within our data as well. Lower and higher income areas were more likely to have lower TRI emissions and to experience increases. The coefficients on homeownership and percent Hispanic, both empowerment variables, support our hypothesis that meaningful toxicity decreases are more likely to happen in communities that are more empowered to influence chemical management decisions.

Our results suggest that the choice of exposure measure, gross air emissions *versus* toxicity-weighted emissions, can greatly impact the type of environmental risk relationship one observes. While this analysis does not capture the impact of the smaller pollution sources and mobile sources, we can tell that the potential health burden from TRI is disproportionately affecting “disadvantaged” areas. From a local perspective, we know that there are “hotspots” where HAP emissions and toxicity have increased over time, communities with large minority populations, lower incomes, and located near major mobile emissions sources.

**Table 2:** OLS and Spatial Regressions for Change in Toxicity-weighted Emissions (1989–2002).

Variables	OLS		Spatial Lag 1		Spatial Lag 2	
	Coefficient	T-Stat	Coefficient	T-Stat	Coefficient	T-Stat
**Percent Hispanic**	−0.422	−2.449 **	−0.286	−1.681 *	0.326	3.498 ***
**Median Household Income**	8.885e-06	2.974 ***	7.592e-06	2.578 ***	5.036e-06	3.147 ***
**Percent Owner-Occupied Housing**	−1.160	−4.759 ***	−0.907	−3.755 ***	−0.382	−2.884 ***
**W-log of Toxicity**			0.222	6.285 ***	−0.049	−2.131 **
**Log of 1989 Toxicity**					−0.910	−67.515 ***
**N**	1942		1942		1942	
**R^2^**	0.017		0.047		0.720	

Significant at the *** 0.01 level; ** 0.05 level; * 0.10 level.

Our models for change in TRI emissions over time are slightly divergent from previous environmental risk models, first in that they are spatial models, and second, that the directionality of the coefficients does not exactly match risk disparity theory. Studies situated in California have consistently found percent minority to be *positively* correlated with exposure, for TRI facility proximity and estimated cancer risk [6,9], where we found the direction of percent Hispanic to be variable, but always significantly correlated with TRI exposure. Characterization of ethnicity seems to vary based on where in the U.S. a study is conducted, and in our case Hispanics comprise the predominant minority group. Our finding regarding poverty mirrors the results of Mennis [18] and Sheppard *et al.* [19], but not Ringquist [20], who attributes the negative correlation between poverty and exposure to a predominance of facilities in working class, middle-income neighborhoods. We found that TRI exposures tend to increase as median income increases, while Bowen *et al.* [21] found median household income to be negatively associated with TRI exposure at the tract level, as did two studies set in California [7,22]. The lack of concurrence among studies is likely due to wide geographic variation, unit of analysis [23], and a significant interaction often observed between ethnicity and income [28]. Low rates of home-ownership were sometimes significantly correlated with higher TRI exposures [24]. Our analysis differs most in the inclusion of 1989 emissions as an explanatory variable, and the use of a spatial lag term. The 1989 emissions are unique to our longitudinal design, while the spatial nature of environmental risk variables is typically accounted for some sort of land use or population density term, or simply not addressed.

### 3.3. A Structural Model of Stakeholder Empowerment

**Figure 2:**
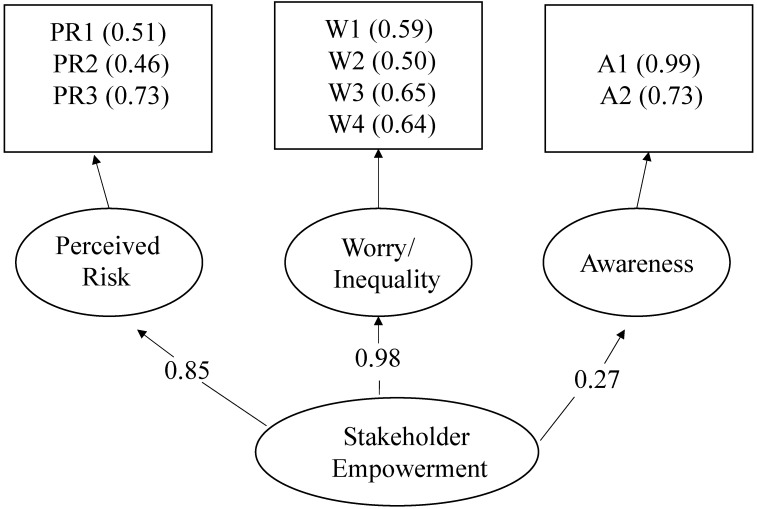
Model for Stakeholder Empowerment—Hierarchical confirmatory factor analysis was used to generate factor loadings for each of the three first order factors (perceived risk, worry/inequality, and awareness), as well as the indicators (inside the boxes). The indicators represent individual survey questions that grouped well under our theoretical framework (See Table 3).

The theoretical underpinnings of social empowerment informed the model presented in Figure 2, where stakeholder empowerment is represented as a second order factor, while worry/concern, perceived risk, and awareness are first order factors. Each factor is comprised of indicators (boxes), which are individual survey questions. The first order factors are derived from theoretically important variable groupings with high reliability.

*Worry*/*inequity* loads most strongly onto empowerment, with a standardized factor weight of 0.98 (α = 0.678). The construct consists of individual feelings of worry or concern over environmental quality, as well as one’s judgment regarding whether poor people are more exposed to dangerous chemicals. Higher scores actually indicate less worry and less belief that the poor are more exposed. Worry & Inequity seem to be influenced by stakeholder empowerment because individuals who feel more empowered to act on behalf of their interests both feel more control over and worry less about environmental risks.

*Perceived risk* also loads strongly on stakeholder empowerment, with a standardized factor weight of 0.84 (α = 0.573). This construct is made up of questions about one’s perceptions of general environmental risk—whether the environment is more polluted than 10 years ago, whether chemicals in the environment cause serious health problems, and how much cancers are caused by exposure to tangible environmental agents. In this case, the variable is reverse-coded and lower scores are important. The lower scores indicate higher perceived risk. Interestingly, although high-risk perceptions fit the model, worry is low. The mechanism here may be attributed to a relationship between empowerment and awareness of risks, although not necessarily accuracy regarding that awareness.

**Table 3:** Correlation matrix and standard deviations of the variables used in the main model.

Variable	Description	Correlation Matrix										
		n	M	SD	PR1	PR2	PR3	W1	W2	W3	W4	A1
**PR1**	Perceived changes in environmental risk	203	1.37	0.66								
**PR2**	Perceived risk: environmental pollution	202	1.68	0.45	0.27 **^†^**							
**PR3**	Perceived risk: environmental cancers	202	1.27	0.59	0.36 **^†^**	0.32 **^†^**						
**W1**	Worry: environmental health risks	203	2.78	0.89	0.29 **^†^**	0.17 *	0.34 **^†^**					
**W2**	Fairness: poor are more exposed	200	1.44	0.64	0.21 **^†^**	0.29 **^†^**	0.37 **^†^**	0.22 **^†^**				
**W3**	Worry: health affected by environment	203	3.03	0.93	0.19 **^†^**	0.26 **^†^**	0.42 **^†^**	0.40 **^†^**	0.33 **^†^**			
**W4**	Worry: frequency of fears	203	2.16	0.84	0.31 **^†^**	0.13	0.39 **^†^**	0.42 **^†^**	0.30 **^†^**	0.39 **^†^**		
**A1**	Awareness: air pollution sources	203	0.94	0.76	0.15 **	0.20 **^†^**	0.00	0.09	0.15	0.20 **^†^**	0.28 **^†^**	
**A2**	Awareness: information sources	203	0.75	0.64	0.06	0.14 *	0.00	0.07	0.13	0.11	0.25 **^†^**	0.72 **^†^**

Pearson correlation coefficients are significant at the * 0.05 level or **^†^** 0.01 level (two-tailed).

*Awareness* loads relatively weakly, with a standardized factor weight of only 0.28 (α = 0.831). It is, however, an important component of stakeholder empowerment, as it captures an outcome that is more closely related to one’s ability to truly utilize TRI data and participate in the chemical management process. This construct is comprised of one’s ability to identify sources of air pollution and the range of resources respondents used to access information about local air pollution sources. *Awareness* is highly correlated to the ability to identify air pollution sources (*r* = 0.93) and number of information sources utilized (*r* = 0.77). It is the most weakly loading of the factors, and is possibly unimportant to one’s policy preferences with regard to air pollution issues [19], but we retain *awareness* in the model, as one cannot participate in the process without first being aware of the program as a resource. The majority of communities in the U.S. face negligible risk from TRI emissions, as facilities tend to be clustered in their distribution [20] and so the empowerment process begins with the ability to identify local risks. Although the indicators of *awareness* deal with general air pollution information and not TRI specifically, it is likely that a similar mechanism is at work. Of those respondents who were aware of TRI or similar programs, whether they had actually looked at TRI data was strongly correlated (*r* = 0.320, *p* < 0.01) with the number of sources of air pollution information they cited.

Overall, *worry/inequity* and *risk perception* get at the intrapersonal aspect of empowerment—one’s sense of control or self-efficacy—but in the context of how they respond to environmental risks. *Risk perception* and *awareness* speak more to the interaction component, which relates to one’s ability to understand the resources available to them (e.g., air pollution information), and more specifically, but unclear from our study, the social and political structures that enable them to act [28,29,30,31,32].

#### 3.3.1. Model Fit

The structural equation model for fits the concept of empowerment reasonably well (Chi-square = 46.634, df = 25, *p* = 0.005, comparative fit index (CFI) = 0.948, root mean square error of approximation (RMSEA) = 0.065). This is the most parsimonious model. We tested other models with up to 14 indicators among the three first order factors, in order to incorporate a broader spectrum of potential influences on empowerment. Variables included income, and further questions related to one’s personal exposure (e.g., “I am exposed to environmental risks simply by living in this community.”) fairness (e.g., “Some communities are treated unfairly when decisions are made about dangerous things in the environment.”), and level of formal education. The resulting overall model fit statistics and Cronbach’s alpha values for individual variable groupings both suggested that the model be simplified (correlation matrix presented in Table 3).

#### 3.3.2. Linking the Model to Outcomes

We were also interested in whether or not the construct of empowerment could be correlated with awareness of the TRI program, reductions in TRI emissions over time, or demographic characteristics of the respondents. We measured the correlation between TRI awareness (*A3*) and the main model to be −0.062 (cov = −0.091, *p* = 0.442). We found no statistically significant association between TRI emission changes (*ObsIncr*) and the empowerment model (*r* = −0.011, cov = −0.006, *p* = 0.891). This was not surprising, as individual empowerment is merely one component of the empowerment process, and change in emissions is not a social/policy outcome directly addressed by the TRI program. There is also an issue of defining social/policy change, in terms of what constitutes a meaningful decline in TRI emissions, and at what geographical level. This study examines clusters of census block groups, but it is possible that individual empowerment has more significantly impacted regional emissions than individual facilities or neighborhoods.

Further evaluating TRI poses an interesting challenge to policy makers in that one of its primary goals, stakeholder empowerment, cannot be directly measured. This analysis, however, begins to construct a framework for understanding one component of empowerment. The three factors, *risk perception*, *worry/inequity*, *and awareness*, each appear to play a role in what we have labeled *individual empowerment*, and this basic structure informs how we might better elicit whether or not TRI is fulfilling its programmatic goals. Although certain components of our model parallel components of psychological empowerment, an important correlate to active participation [29], the questions were focused on responses to environmental quality and the specific issue of air pollution.

With an individual empowerment framework created, we must next turn our focus to the process by which empowerment affects social/policy changes, along the vein of TRI use, action, and subsequent emissions changes. The measured level of TRI awareness did not have any meaningful correlation with our model, nor did measures of emissions change. This is likely a result of the complexity of the steps between the condition of empowerment and the environmental outcome. In fact, while feelings of personal control and perceptions of risk may influence whether or not an individual will actually change their behaviors in response to environmental threats, the link between personal concern for the environment and action tends to be weak [33,34,35]. TRI awareness may work better as an integral component of the model, as in the alternate model we tested, where it can function as an indicator for *awareness.* A larger sample size and a continuous scale of emissions changes could more accurately capture the relationship between knowledge of TRI and other aspects of stakeholder empowerment, and perhaps show a stronger link with air pollution awareness variables. It would also be useful to quantify how a model of empowerment is influenced by demographic characteristics. An attempt to correlate the model with age and percent minority resulted in an inadmissible model solution, though we know risk perception and individual risk judgments to follow socio-demographic patterns [27].

Individuals must then be able to interpret emissions data and quantify health risks to establish grounds for concern, and access to the appropriate structure or agency for translating knowledge into action, and exercising empowerment. These subsequent steps from empowerment to social change deal more with intermediate outcomes than with processes. This distinction is important because they comprise two different elements of empowerment theory [34], and because the outcomes are more difficult to empirically measure and are closely tied to external influences. While our study found several individuals who had taken action, such as contacting an environmental organization, further detail would have been difficult to obtain without using a narrative approach. The outcome of emissions decreases, while more easily measured, is likely impacted by economic trends, industry-level decisions, existing regulations, and various other factors that are external to stakeholder empowerment.

## 4. Conclusions

This research raises some important questions about tracking environmental risk disparity over time and about our ability to gauge the efficacy of policies designed to reduce pollution—do they help everyone or do they exacerbate the problem of inequitable distribution of environmental risk? Our results support the notions that (i) environmental exposures such as TRI emissions are clustered throughout Los Angeles, and possibly, similar urban areas; and (ii) improvements in environmental quality due to TRI emissions and toxicity decreases tend to benefit populations that are more economically and socially empowered. We find that the exposure measure and choice of geographic region both greatly impact the nature of the environmental risk relationship, which is why we observe such wide disparities among environmental risk analyses, and why some of our findings run counter to those of previous studies. We suggest that focusing on changes in environmental exposures over time allows a more targeted and meaningful discussion of the *processes* that lead to disproportionate impacts on disadvantaged communities.

TRI was designed in a good faith effort to promote access and openness in a regulatory realm traditionally characterized by its secrecy and inaccessibility. In practice, however, it is unclear whether the program has meaningfully impacted public empowerment. The model put forth in this paper begins to address how such a concept can be measured by examining certain individual characteristics it influences. We found statistically supported data groupings, *risk perception, worry/inequity,* and *awareness,* which provide some empirical support for our theoretical model of individual stakeholder empowerment. We were able to derive a parsimonious model with relatively good fit. Risk perception and worry are the most strongly loading concepts, which is consistent with previous findings regarding the role of personal risk estimation and feelings of control in response to environmental hazards.

This research is particularly timely given the recent debacle surrounding the discovery of massive long-term lead (Pb) arsenic, cadmium and other hazardous emissions from the Exide lead-acid battery recycling facility in the Vernon district located approximately 8 km southeast of downtown Los Angeles. In March 2015, Exide agreed to a settlement in a federal criminal investigation to shut down the facility, and to commit $50 million for cleaning up the facility and surrounding neighborhoods, and another $9 million trust fund for decontaminating 216 nearby residences in the Boyle Heights neighborhood and the City of Maywood, many with low-income families [36]. By providing a starting point for investigating the structure of stakeholder empowerment and developing a method for evaluating information-based environmental regulations, this research underscores the importance of the U.S. EPA’s commitment to Environmental Justice through the EJ 2020 Action Agenda that advances the accomplishments of the prior Plan EJ 2014 [37]. EJ 2020 is designed to support the federal agency’s advancement of environmental justice through its programs, policies and activities, including cross-agency strategy on transformative environmental interventions in environmentally overburdened, underserved, and economically distressed communities [38].

## References

[1] Kulkarni S.P. (2000). Environmental ethics and information asymmetry among organizational stakeholders. J. Bus. Ethics.

[2] Hearne S.A. (1996). Tracking toxics: Chemical use and the public’s “right-to-know”. Environment.

[3] U.S. EPA Toxic Release Inventory, 2015. http://www.epa.gov/tri/whatis.htm.

[4] Perkins D.D. (1995). Speaking truth to power: Empowerment ideology as social intervention and policy. Am. J. Commun. Psychol..

[5] NFIB (National Federation of Independent Businesses), 2015. http://www.nfib.com.

[6] U.S. Government (2005). U.S. EPA—Toxics release inventory burden reduction proposed rule. Fed. Register.

[7] Pastor M., Morello-Frosch R., Sadd J.L. (2005). The air is always cleaner on the other side: Race, space, and ambient air toxics exposures in California. J. Urban Aff..

[8] Anselin L. (1995). Local indicators of spatial association—LISA. Geogr. Anal..

[9] Mennis J.L., Jordan L. (2005). The distribution of environmental equity: Exploring spatial nonstationarity in multivariate models of air toxic releases. Ann. Assoc. Am. Geog..

[10] Boer J.T., Pastor M., Sadd J.L., Snyder L.D. (1997). Is there environmental racism? The demographics of hazardous waste in Los Angeles County: Research on the environment. Soc. Sci. Quart..

[11] De Marchi S., Hamilton J.T. (2006). Assessing the accuracy of self-reported data: An evaluation of the toxics release inventory. J. Risk Uncertain..

[12] EIP (Environment Integrity Project), 2015. http://environmentalintegrity.org.

[13] Natan T.E., Miller C.G. (1998). Peer reviewed: Are toxics release inventory reductions real?. Policy Anal..

[14] U.S. EPA The Toxics Release Inventory in Action: Media, Government, Business, Community and Academic Uses of TRI Data, 2013. http://www2.epa.gov/sites/production/files/documents/tri_in_action_final_report_july_2013.pdf.

[15] U.S. EPA TRI Explorer, 2015. http://iaspub.epa.gov/triexplorer/tri_release.chemical.

[16] Morello-Frosch R., Jesdale B.M. (2006). Separate and unequal: Residential segregation and estimated cancer risks associated with ambient air toxics in US metropolitan areas. Environ. Health Persp..

[17] Morello-Frosch R., Pastor M., Sadd J. (2001). Environmental justice and southern California’s “riskscape”: The distribution of air toxics exposures and health risks among diverse communities. Urban. Aff. Rev..

[18] Mennis J. (2002). Using geographic Information systems to create and analyze statistical surfaces of population and risk for environmental justice analysis. Soc. Sci. Quart..

[19] Sheppard E., Leitner H., McMaster R.B., Tian H. (1999). GIS-based measures of environmental equity: Exploring their sensitivity and significance. J. Expo. Anal. Env. Epidemiol..

[20] Ringquist E.J. (1997). Equity and the distribution of environmental risk: The case of TRI facilities. Soc. Sci. Quart..

[21] Bowen M.W., Salling M.J., Haynes K.E., Cyran E.J. (1995). Toward environmental justice: Spatial equity in Ohio and Cleveland. Ann. Assoc. Am. Geog..

[22] Pastor M., Sadd J.L., Morello-Frosch R. (2004). Waiting to inhale: The demographics of toxic air release facilities in 21st-century California. Soc. Sci. Quart..

[23] Dolinoy D.C., Miranda M.L. (2004). GIS modeling of air toxics releases from TRI-reporting and non-TRI-reporting facilities: Impacts for environmental justice. Environ. Health Persp..

[24] Apelberg B.J., Buckley T.J., White R.H. (2005). Socioeconomic and racial disparities in cancer risk from air toxics in Maryland. Environ. Health Persp..

[25] Macey G.P., Her X., Reibling E.T., Ericson J. (2001). An investigation of environmental racism claims: Testing environmental management approaches with a geographic information system. Environ. Manag..

[26] Boone C.G., Modarres A. (1999). Creating a toxic neighborhood in Los Angeles County a historical examination of environmental inequity. Urban. Aff. Rev..

[27] Gerber B.J., Neeley G.W. (2005). Perceived risk and citizen preferences for governmental management of routine hazards. Policy Stud. J..

[28] Perlin S.A., Wong D., Sexton K. (2001). Residential proximity to industrial sources of airpollution: Interrelationships among race, poverty, and age. J. Air Waste Manag. Assoc..

[29] Maantay J.A., McLafferty S. (2011). Geospatial Analysis of Environmental Health.

[30] Speer P.W., Peterson N.A. (2000). Psychometric properties of an empowerment scale: Testing cognitive, emotional and behavioural domains. Soc. Work Res..

[31] Zimmerman M.A., Rappaport J. (1988). Citizen participation, perceived control, and psychological empowerment. Am. J. Commun. Psychol..

[32] Fransson N., Garling T. (1999). Environmental concern: Conceptual definitions, measurement methods, and research findings. J. Environ. Psychol..

[33] Bickerstaff K. (2004). Risk perception research: Socio-cultural perspectives on the public experience of air pollution. Environ. Int..

[34] Perkins D.D., Zimmerman M.A. (1995). Empowerment theory, research, and application. Am. J. Commun Psychol..

[35] Vaughan E. (1995). The significance of socioeconomic and ethnic diversity for the risk communication process. Risk Anal..

[36] The United States Attorney—Central District of California, 2015. http://www.justice.gov/usao/cac/Pressroom/2015/027.html.

[37] U.S. EPA (2015). Environmental Justice. http://www.epa.gov/environmentaljustice/ej2020/.

[38] U.S. EPA Draft EJ 2020 Action Agenda Framework, 2015. http://www.epa.gov/environmentaljustice/resources/policy/ej2020/draft-framework.pdf.

